# Intraoperative Driving Pressure and Postoperative Pulmonary Complications Following Cardiac Surgery: A Prospective Observational Study †

**DOI:** 10.3390/medicina62061167

**Published:** 2026-06-16

**Authors:** Canan Yılmaz, Filiz Ata, Selimcan Yırtımcı, Eralp Çevikkalp, Emre Ulusoy, Ümran Karaca, Ayşe Neslihan Balkaya, Tuğba Onur, Abdulkadir İskender, Mehmet Gamlı

**Affiliations:** 1 Department of Anesthesiology and Reanimation, University of Health Sciences, Bursa Yüksek İhtisas Training and Research Hospital, Yıldırım 16310, Bursa, Türkiyedoktor-t@hotmail.com (T.O.); 2 Department of Anesthesiology and Reanimation, University of Health Sciences, Bursa City Hospital, Nilüfer 16250, Bursa, Türkiye

**Keywords:** driving pressure, cardiac surgery, cardiopulmonary bypass, postoperative pulmonary complications, lung-protective ventilation, mechanical ventilation

## Abstract

*Background and Objectives*: Postoperative pulmonary complications (PPCs) remain an important cause of morbidity after cardiac surgery. Driving pressure (DP), defined as the difference between plateau pressure and positive end-expiratory pressure, has been proposed as a bedside marker of respiratory system mechanics during lung-protective ventilation. However, its relationship with PPCs in patients undergoing cardiac surgery with cardiopulmonary bypass (CPB) remains uncertain. This study aimed to evaluate the association between intraoperative DP and PPCs following CPB-supported cardiac surgery. *Materials and Methods*: This single-center prospective observational study included 99 adult patients undergoing elective cardiac surgery with CPB. All patients were ventilated using a standardized lung-protective strategy with a tidal volume of 6 mL/kg predicted body weight and a fixed PEEP of 5 cmH_2_O. Patients were categorized according to intraoperative DP as Group I (DP < 13 cmH_2_O, *n* = 66) and Group II (DP ≥ 13 cmH_2_O, *n* = 33). The primary outcome was a composite PPC endpoint, defined as the occurrence of at least one EPCO-defined pulmonary complication during the postoperative hospital stay. Multivariable logistic regression was performed to assess whether pre-CPB DP was independently associated with PPCs after adjustment for body mass index, CPB time, and age. *Results*: Patients with DP ≥13 cmH_2_O had higher post-CPB and ICU-admission lactate concentrations. Pneumothorax, pleural effusion, atelectasis, CPAP requirement, and prolonged mechanical ventilation were more frequent in the elevated-DP group. Mechanical ventilation duration, ICU stay, and hospital stay were also longer in this group. Composite PPCs occurred in 41 patients (41.4%). Although higher pre-CPB DP showed a non-significant trend toward increased PPC risk in univariable analysis (OR 1.121, 95% CI 0.988–1.273; *p* = 0.077), it was not independently associated with the composite PPC endpoint after adjustment (adjusted OR 1.091, 95% CI 0.952–1.251; *p* = 0.212). In contrast, higher pre-CPB DP was significantly associated with prolonged postoperative ventilation and longer mechanical ventilation, ICU, and hospital stay durations. *Conclusions*: Elevated intraoperative DP was associated with a higher unadjusted burden of PPCs and delayed postoperative recovery after CPB-supported cardiac surgery. However, pre-CPB DP was not an independent predictor of the composite PPC endpoint after adjustment for relevant confounders. These findings suggest that DP may serve as a clinically useful marker of impaired respiratory mechanics and postoperative vulnerability rather than as an independent causal determinant of PPCs.

## 1. Introduction

Postoperative pulmonary complications (PPCs) remain an important driver of morbidity and mortality after cardiac procedures. Reported incidence rates range from 10% to 25% and include atelectasis, pneumonia, pleural effusion, hypoxemia, and acute respiratory distress syndrome (ARDS). Such events are associated with longer intensive care unit (ICU) and hospital stays, higher treatment costs, and delayed patient recovery [1].

Lung-protective mechanical ventilation aims to limit ventilator-induced injury and is based on three central principles: low tidal volume, a clinically appropriate level of positive end-expiratory pressure (PEEP), and avoidance of excessive plateau pressures. In the cardiac surgical setting, several investigations have suggested that protective ventilation may reduce the burden of PPCs [2]. Nevertheless, the published literature is not uniform; some trials have reported substantial benefits, whereas others have found only marginal or non-significant effects on pulmonary outcomes [3,4]. More recently, driving pressure (DP) has gained prominence as a key element within the protective-ventilation framework. By definition, DP is the difference between plateau airway pressure and applied PEEP, and this parameter is thought to reflect the cyclic mechanical load imposed on the respiratory system [3]. As such, DP can be regarded as a bedside surrogate of respiratory system mechanics. In ARDS, sustained elevations in DP have been associated with worse outcomes, including higher mortality and a greater likelihood of ventilator-induced lung injury [3,4]. Evidence also extends beyond ARDS: several reports indicate that high intraoperative DP is associated with subsequent PPCs in patients receiving general anesthesia for surgery [5].

Despite this, only a small number of studies have specifically addressed DP in cardiac surgical patients, and the available data remain limited [5,6]. Cardiac surgery represents a uniquely vulnerable setting because of CPB-related effects, transient cessation of pulmonary perfusion, systemic inflammatory activation, perioperative volume shifts, and changes in cardiac performance [7]. Together, these factors complicate intraoperative ventilation management and make it difficult to evaluate protective strategies. Consequently, the association between intraoperative DP and pulmonary outcomes after cardiac surgery remains incompletely defined, and further investigation is warranted.

We hypothesized that lower intraoperative DP during CPB-supported cardiac surgery would be associated with more favorable postoperative pulmonary outcomes. Our primary objective was therefore to evaluate the association between intraoperative DP and PPC occurrence. Secondary objectives included examining its relationship with prolonged postoperative ventilation, ICU and hospital length of stay, and 30-day mortality.

## 2. Methods

### 2.1. Study Design

This investigation was conducted as a single-center, prospective observational study. Following approval from the Tertiary University Hospital Clinical Research Ethics Committee (Approval No: 2011-KAEK-25, 2020/01-20; date of approval: 2020), perioperative records of patients scheduled for elective open-heart surgery between 1 February and 1 August 2020 were systematically collected and analyzed. The study was approved by the appropriate ethics committee before patient enrollment began, and all procedures were conducted in accordance with the principles set forth in the Declaration of Helsinki. All patients provided written informed consent for participation in the study and for publication of their anonymized data. The manuscript was prepared in accordance with the Strengthening the Reporting of Observational Studies in Epidemiology (STROBE) statement.

### 2.2. Study Population and Patient Selection

Eligibility criteria included adults aged 18 years or older, classified as ASA physical status II–IV, with a body mass index (BMI) of 40 kg/m^2^ or below, scheduled for elective isolated coronary artery bypass grafting with cardiopulmonary bypass (CPB).

Several exclusion criteria were applied. From a respiratory standpoint, patients with chronic obstructive pulmonary disease, asthma, obstructive sleep apnea, recent pulmonary infection (within one month), mechanical ventilation or extracorporeal membrane oxygenation support during the preceding two months, single-lung ventilation requirements, or an FEV_1_/FVC ratio < 70% on pulmonary function testing were not included. Preoperative respiratory insufficiency was defined as oxygen saturation below 90%, PaO_2_ under 60 mmHg, a PaO_2_/FiO_2_ ratio less than 300, or PaCO_2_ exceeding 45 mmHg on arterial blood gas analysis. Cardiac exclusion criteria comprised an ejection fraction below 30%, severe pulmonary hypertension, active endocarditis, ongoing inotropic support before surgery, cardiogenic shock, and intraoperative use of an intra-aortic balloon pump. Systemic exclusions included BMI above 40 kg/m^2^, insulin-treated diabetes, dialysis-dependent renal failure, advanced hepatic disease, neuromuscular disorders, and previous lung or heart transplantation. In addition, intraoperative withdrawal occurred if persistent respiratory acidosis (PaCO_2_ > 45 mmHg) or inadequate oxygenation (SpO_2_ < 90% or PaO_2_/FiO_2_ < 100 with FiO_2_ > 80%) developed despite optimized ventilator adjustments.

### 2.3. Anesthetic Management

Premedication consisted of intravenous midazolam (Dormicum^^®^^; Roche, Basel, Switzerland) 0.03 mg/kg. Anesthesia was induced using slow boluses of thiopental (Pental Sodyum^®^, İ.E. Ulagay İlaç Sanayii Türk A.Ş., Istanbul, Türkiye) (3–5 mg/kg), fentanyl (Fentanyl-Hameln^®^; Hameln Pharma, Hameln, Germany) (1–2 µg/kg), and rocuronium (Esmeron^®^, N.V. Organon, Oss, the Netherlands) (0.6–0.9 mg/kg). Maintenance was achieved with sevoflurane (Sevorane^®^, AbbVie S.r.l., Campoverde di Aprilia, Italy) at MAC 1.0 (oxygen-to-air ratio 1:1) using volume-controlled continuous mandatory ventilation (Drägerwerk AG & Co. KGaA, Lübeck, Germany) (VC-CMV) and a continuous remifentanil (Ultiva^®^, GlaxoSmithKline Manufacturing S.p.A., Parma, Italy) infusion (0.1–0.5 µg/kg/min). Remifentanil dosing was adjusted according to hemodynamic responses across the three CPB phases—pre-bypass, bypass, and post-bypass—to maintain intraoperative hemodynamic stability. Postoperative analgesia followed an opioid-sparing multimodal protocol: at the conclusion of surgery, an experienced anesthesiologist performed bilateral parasternal intercostal fascial plane (PIFP) blocks (20 mL per side), while the operating surgeon supplemented the regimen with local infiltration of the drain insertion sites. Scheduled systemic agents included intravenous paracetamol 1 g every six hours and tenoxicam 20 mg once daily, and intravenous morphine (0.05 mg/kg) was reserved as rescue medication for numeric rating scale scores of 4 or higher.

### 2.4. Ventilation Protocol

A uniform lung-protective ventilation strategy was applied in all patients before and after cardiopulmonary bypass (CPB). After induction of general anesthesia and tracheal intubation, patients were ventilated in volume-controlled mode with a tidal volume of 6 mL/kg predicted body weight, a fixed positive end-expiratory pressure (PEEP) of 5 cmH_2_O, and an inspiratory-to-expiratory ratio of 1:2. Apart from FiO_2_ adjustment, ventilator settings were kept unchanged throughout the pre- and post-CPB periods. FiO_2_ was titrated to remain below 80% whenever possible, with the aim of maintaining PaO_2_ between 200 and 250 mmHg. Hematocrit was maintained at ≥24%.

Driving pressure (DP) was calculated as plateau pressure minus PEEP. Plateau pressure was measured during volume-controlled ventilation using an end-inspiratory hold maneuver under fully controlled mechanical ventilation, with no visible spontaneous respiratory effort. Measurements were obtained after stabilization of ventilatory and hemodynamic conditions. DP was recorded at two predefined intraoperative time points: before initiation of CPB and after separation from CPB, following resumption of mechanical ventilation and completion of the recruitment maneuver.

Mechanical ventilation was discontinued during CPB according to the institutional cardiac anesthesia protocol. After separation from CPB and re-expansion of the lungs, mechanical ventilation was resumed using the same lung-protective settings. A standardized recruitment maneuver was then performed by increasing airway pressure to 40 cmH_2_O for at least seven seconds with FiO_2_ maintained below 80%, provided that the patient was hemodynamically stable. The same protective ventilation approach was continued in the postoperative intensive care unit.

Based on previously published thresholds, a DP value of ≥13 cmH_2_O was considered elevated [8,9]. Patients were therefore categorized into two groups according to intraoperative DP: Group I, DP < 13 cmH_2_O, and Group II, DP ≥ 13 cmH_2_O. DP was also evaluated as a continuous variable in additional analyses to avoid overreliance on dichotomization and to explore a potential graded association with postoperative pulmonary complications.

### 2.5. Extubation Protocol

All patients were monitored in the postoperative ICU. Extubation was considered when the following conditions were met: satisfactory gas exchange (PaO_2_/FiO_2_ ≥ 150 with FiO_2_ ≤ 0.40, PEEP ≤ 5 cmH_2_O, minute ventilation ≤15 L/min); chest imaging that was stable or improving and free of excessive secretions; the patient’s ability to sustain spontaneous breathing with little or no respiratory support; effective coughing and intact upper-airway reflexes; hemodynamic stability without significant vasopressor requirements; absence of active myocardial ischemia or pulmonary edema; and a Glasgow Coma Scale score of 8 or higher.

### 2.6. Data Collection and Outcome Parameters

The following parameters were documented: total mechanical ventilation time, time to extubation, prolonged mechanical ventilation defined as >24 h, CPAP requirement, reintubation events, occurrence of respiratory failure, and PPCs. PPCs were classified using the European Perioperative Clinical Outcome (EPCO) consensus definitions and were captured prospectively from the operating-room day until hospital discharge through daily ward rounds led by the same medical team. The primary outcome was a composite postoperative pulmonary complication endpoint, defined as the occurrence of at least one EPCO-defined pulmonary complication during the postoperative hospital stay. Individual PPC components, including atelectasis, pneumonia/respiratory infection, pleural effusion, pneumothorax, respiratory failure, CPAP requirement, reintubation, and prolonged mechanical ventilation, were also recorded and analyzed separately as secondary or exploratory outcomes. Postoperative pulmonary complications were defined according to the European Perioperative Clinical Outcome (EPCO) consensus definitions. Atelectasis was defined as lung opacification detected on chest radiography, computed tomography, and/or thoracic ultrasonography, accompanied by displacement of the mediastinum, hilum, or hemidiaphragm toward the affected area and compensatory overinflation of adjacent non-atelectatic lung. Pneumonia/respiratory infection was defined as clinically suspected respiratory infection requiring antibiotic treatment and accompanied by at least one of the following findings: new or changed sputum, new or changed pulmonary opacities, fever, or leukocytosis. Pleural effusion was defined as chest radiographic evidence of blunting of the costophrenic angle, loss of the sharp silhouette of the ipsilateral hemidiaphragm in the upright position, displacement of adjacent anatomical structures, or, in the supine position, a hazy opacity in one hemithorax with preserved vascular markings. Pneumothorax was defined as the presence of air in the pleural space, characterized radiologically by an area without vascular markings surrounding the visceral pleura.

Standard posteroanterior chest radiographs were obtained for every patient within the first 24 h after surgery, generally at ICU admission. When clinical deterioration was observed—such as refractory hypoxemia (SpO_2_ < 90% on supplemental oxygen), new-onset respiratory distress, hemodynamic instability, or unexpectedly prolonged ventilation—an additional radiograph was ordered without delay at the discretion of the attending intensivist or anesthesiologist, regardless of the time since the previous study. All postoperative imaging was jointly evaluated by the attending intensivist and the radiology team, who were blinded to patient group allocation, and the findings were recorded in the medical record. When chest radiographs were inconclusive, additional assessment was carried out using thoracic computed tomography or bedside lung ultrasound. ICU stay, hospital stay, and 30-day mortality were also analyzed.

### 2.7. Statistical Analysis

Sample size was determined a priori using G*Power (v3.1.9.7, Heinrich-Heine-Universität Düsseldorf, Germany). The primary endpoint was the rate of PPCs. Drawing on previously published series and our own preliminary observations, the anticipated PPC frequency was set at 20% in the low-DP cohort (<13 cmH_2_O) and 50% in the high-DP cohort (≥13 cmH_2_O), corresponding to a Cohen’s h effect size of 0.52. With a two-sided test for two independent proportions, an alpha of 0.05, and power (1 − β) of 0.80, the minimum required sample was 90 patients. To account for potential exclusions and missing data, a target enrollment of approximately 99 patients was adopted, and 99 patients were ultimately analyzed.

Continuous variables are reported as mean ± standard deviation when normally distributed and as median with interquartile range (IQR) when not normally distributed; categorical variables are reported as counts and percentages. The Kolmogorov–Smirnov test was used to assess normality. Categorical comparisons relied on the chi-square test or Fisher’s exact test, as appropriate; continuous variables were compared with the Student t test or, for non-normally distributed variables (e.g., duration of mechanical ventilation and intensive care unit and hospital length of stay), the Mann–Whitney U test.

Factors independently associated with PPCs were assessed by multivariable binary logistic regression using the enter method. Rather than using data-driven variable selection, a small set of clinically relevant covariates was pre-specified; the number of predictors was deliberately constrained by the number of outcome events (41 PPC events among 99 patients) to maintain approximately ten events per variable and limit the risk of overfitting. The model included pre-CPB driving pressure together with body mass index, cardiopulmonary bypass (pump) time, and age. Because cardiopulmonary bypass time and aortic cross-clamp time were strongly collinear (r = 0.84), only bypass time was retained. Associations are expressed as odds ratios (OR) and adjusted odds ratios (aOR) with 95% confidence intervals (CI), per 1-unit increase for continuous predictors. For secondary outcomes, the association of pre-CPB driving pressure was assessed by univariable logistic regression for binary endpoints (prolonged postoperative ventilation and 30-day mortality) and by linear regression (β coefficient per 1 cmH_2_O), corroborated by Spearman rank correlation, for continuous endpoints. A two-sided *p* value < 0.05 was considered statistically significant. All analyses were performed using IBM SPSS Statistics for Windows, Version 32.0.0.0 (IBM Corp., Armonk, NY, USA).

## 3. Results

### 3.1. Baseline Characteristics and Clinical Variables

**Figure 1:**
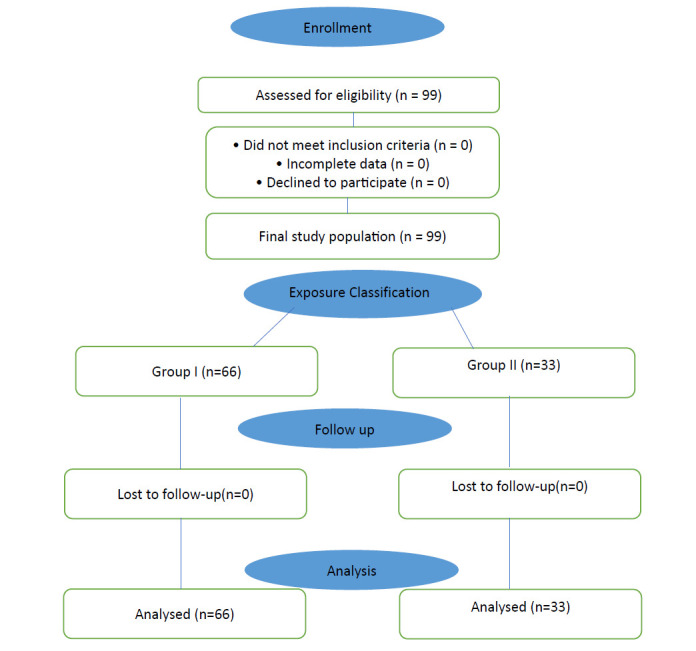
Study Flow Diagram.

During the study period, 102 patients were initially screened. After exclusion criteria were applied and incomplete records were removed, 99 patients formed the final analysis cohort (Figure 1). Of these, 66 were assigned to Group I (DP < 13 cmH_2_O) and 33 to Group II (DP ≥ 13 cmH_2_O). With the exception of BMI—which was higher in Group II (*p* = 0.037)—baseline demographics and preoperative clinical variables did not differ meaningfully between the groups (Table 1).

### 3.2. Intraoperative Data and Driving Pressure

CPB time, aortic cross-clamp time, total anesthesia time, and surgical duration were comparable between cohorts (*p* > 0.05). DP values during the pre-CPB phase were significantly higher in Group II than in Group I, and the same pattern was observed post-CPB (both *p* < 0.001, Table 2).

### 3.3. Perioperative Hemodynamics and Infection Markers

No meaningful between-group differences were observed in perioperative hemodynamic parameters or postoperative infection-related markers (*p* > 0.05, Table 3).

### 3.4. Arterial Blood Gas Analysis

Arterial blood gas analysis demonstrated higher lactate levels in Group II compared with Group I during the post-CPB period and at ICU admission. Post-CPB lactate was 3.14 ± 1.34 mmol/L in Group II and 2.60 ± 1.03 mmol/L in Group I (*p* = 0.031). Similarly, lactate levels at ICU admission were higher in Group II than in Group I (3.21 ± 1.34 vs. 2.59 ± 1.14 mmol/L, *p* = 0.018).(Table 4) No statistically significant intergroup differences were observed in the remaining arterial blood gas parameters, including pH, PaCO_2_, PaO_2_, base excess, hematocrit, and lactate values at other time points.

**Table 4:** Arterial blood gas analysis parameters.

	Group I (*n* = 66)	Group II (*n* = 33)	*p*
Post-CPB lactate (mmol/L)	2.60 ± 1.03	3.14 ± 1.34	0.031 *
ICU admission lactate (mmol/L)	2.59 ± 1.14	3.21 ± 1.34	0.018 *

Values are presented as mean ± standard deviation. CPB: cardiopulmonary bypass; ICU: intensive care unit. * *p* < 0.05.

### 3.5. Postoperative Pulmonary Complications and Clinical Outcomes

Postoperative complications and clinical outcomes are summarized in Table 5. Pneumothorax was more frequent in Group II than in Group I (*p* = 0.035). Pleural effusion was diagnosed in 42.42% of patients in Group II compared with 19.70% in Group I (*p* = 0.017), and atelectasis was identified in 39.39% versus 18.18% of patients, respectively (*p* = 0.022). The need for CPAP support was substantially higher in Group II (*p* = 0.002), as was the proportion of patients requiring prolonged mechanical ventilation beyond 24 h (27.27% vs. 7.58%, *p* = 0.011). Postoperative ventilation duration, ICU stay, and overall hospital stay were each longer in Group II than in Group I (*p* < 0.001, *p* = 0.002, and *p* = 0.004, respectively). The frequencies of bronchospasm, pneumonia, reintubation, and 30-day mortality did not differ significantly between groups (*p* > 0.05).

Postoperative pulmonary complications (PPCs) occurred in 41 of the 99 patients (41.4%). On univariable logistic regression, a higher pre-CPB driving pressure showed a non-significant trend toward an increased risk of PPCs (OR 1.121, 95% CI 0.988–1.273; *p* = 0.077). To test whether this association was independent, pre-CPB driving pressure was entered into a multivariable binary logistic regression model together with a pre-specified set of clinically relevant covariates—body mass index, cardiopulmonary bypass (pump) time, and age; the number of predictors was constrained by the number of outcome events (41 events) to maintain approximately ten events per variable (Table 6).

After adjustment, pre-CPB driving pressure was not independently associated with PPCs. Although a slight upward trend in PPC risk was observed with rising pre-CPB driving pressure, the relationship did not reach statistical significance (adjusted OR 1.091, 95% CI 0.952–1.251; *p* = 0.212). None of the covariates was independently associated with the outcome, including body mass index (aOR 1.052, 95% CI 0.953–1.162; *p* = 0.314), cardiopulmonary bypass time (aOR 1.002, 95% CI 0.990–1.014; *p* = 0.792), and age (aOR 1.022, 95% CI 0.979–1.067; *p* = 0.322).

The relationship between pre-CPB driving pressure and secondary outcomes is summarized in Table 7. In contrast to the composite PPC endpoint, higher pre-CPB driving pressure was significantly associated with prolonged postoperative ventilation (OR 1.314 per cmH_2_O, 95% CI 1.101–1.568; *p* = 0.003). Each 1 cmH_2_O increase in pre-CPB driving pressure was associated with longer durations of mechanical ventilation (β = 2.07 h, 95% CI 0.99–3.15; *p* < 0.001), ICU stay (β = 0.16 days, 95% CI 0.07–0.26; *p* = 0.001), and hospital stay (β = 0.23 days, 95% CI 0.04–0.42; *p* = 0.019). Pre-CPB driving pressure was not significantly associated with 30-day mortality (OR 1.141, 95% CI 0.960–1.357; *p* = 0.135).

## 4. Discussion

In this prospective observational analysis of patients undergoing cardiac surgery with CPB, 33.3% of those managed with conventional lung-protective ventilation and fixed PEEP exhibited a DP of 13 cmH_2_O or higher. Within this high-DP subset, post-CPB and ICU-admission lactate concentrations were higher and PPCs occurred more frequently. In addition, mechanical ventilation, ICU stay, and hospital stay were each prolonged when DP was elevated. It should be emphasized that although patients with elevated DP had a higher unadjusted burden of PPCs, pre-CPB DP was not independently associated with the composite PPC endpoint after adjustment for BMI, CPB time, and age. Therefore, the present findings should not be interpreted as evidence of a causal or independent effect of DP on the development of PPCs. Rather, elevated DP may reflect impaired respiratory system mechanics, reduced compliance, or greater perioperative vulnerability in patients undergoing CPB-supported cardiac surgery. Nevertheless, the significant associations observed between pre-CPB DP and secondary recovery outcomes, including prolonged postoperative ventilation and longer ICU and hospital stays, suggest that DP may still have prognostic value as a simple bedside marker of delayed postoperative recovery.

The combination of low tidal volumes with fixed PEEP is a widely adopted lung-protective approach during cardiac surgery with CPB. However, this uniform approach can mask important between-patient differences in respiratory mechanics, especially variations in static compliance. When PEEP is held constant, DP becomes largely dependent on each patient’s static lung compliance. The emergence of an elevated DP under standardized tidal volume and PEEP settings may therefore indicate a mismatch between the chosen ventilatory strategy and the actual mechanical behavior of the lung parenchyma or chest wall. Mechanistically, DP corresponds to the cyclic pressure change imposed on the respiratory system during each breath and is the arithmetic difference between plateau pressure and PEEP [4,10]. A high DP may signal reduced lung compliance and has been associated with susceptibility to ventilator-induced lung injury (VILI), potentially through phenomena such as alveolar overdistension and repeated opening and collapse of unstable units [11]. In a meta-analysis involving surgical patients receiving general anesthesia, every 1 cmH_2_O rise in DP was associated with a 1.16-fold increase in PPC risk [3]. The LAS VEGAS investigation reported concordant findings, demonstrating an association between elevated DP and PPCs in both open and laparoscopic abdominal procedures [12].

Even so, the protective effect of a low DP is not universally accepted. The IMPROVE-2 trial showed that targeting a DP under 13 cmH_2_O did not reduce PPC rates after emergency abdominal surgery, and several observational analyses in open abdominal procedures have likewise been unable to demonstrate an independent association between DP and PPCs [13,14]. Such inconsistencies may stem from dynamic shifts in chest wall elastance during abdominal procedures—particularly pneumoperitoneum and patient positioning—which can distort DP readings. Excessive PEEP can also increase mechanical power and may promote overdistension rather than effective recruitment, thereby contributing to iatrogenic injury. During patient-tailored PEEP titration, an increase in PEEP should ideally be accompanied by a decrease in DP. Neto and colleagues reported that when DP increased despite higher PEEP, the risk of PPC was increased [3].

Disentangling the contribution of DP to PPCs in cardiac surgery is complicated by numerous confounders, which may produce variable estimates. Once a sternotomy has been completed, chest wall mechanics may remain relatively stable compared with abdominal cases, while pulmonary changes related to CPB may occur mainly at the parenchymal level. DP may therefore provide a practical bedside measure of respiratory system mechanics in this population. Consistent with this concept, elevated DP has been identified as an independent predictor of PPCs in heart-transplant recipients [15]. In our cohort, patients with elevated DP had a higher unadjusted PPC burden. In contrast to abdominal procedures, the inflammatory response triggered by CPB, together with increased capillary permeability and ischemia–reperfusion injury, may reduce lung compliance and predispose patients to higher DP under unchanged ventilator settings. In keeping with this mechanism, higher DP values were observed during the post-CPB phase.

Our data suggest that, in operations characterized by inflammatory and ischemic pulmonary stress—such as those involving CPB—a DP at or above 13 cmH_2_O may identify patients with less favorable respiratory mechanics and higher postoperative vulnerability. A fixed PEEP regimen may not provide adequate recruitment in selected patients. In such patients, the combination of fixed tidal volume and fixed PEEP may be associated with overdistension or increased mechanical power exposure rather than the intended recruitment effect.

Higher BMI, shorter stature, and female sex have all been described as predictors of elevated DP. Each 1 kg/m^2^ increase in BMI has been associated with roughly a 0.35 cmH_2_O rise in DP, while each 1 cm reduction in height has been associated with a 0.01 cmH_2_O increase in DP [14]. Greater airway resistance in shorter patients and women may contribute to higher airway pressures and consequently higher DP. In our analysis, BMI was higher in the high-DP cohort. Increased BMI may reduce respiratory system compliance through increased chest wall elastance, which may in turn increase DP.

Elevated DP may indicate increased cyclic load on the functional lung tissue—the so-called “baby lung”—and has been hypothesized to be related to biotrauma through systemic release of pro-inflammatory cytokines and mediators [16,17]. Clinical and laboratory studies have reported associations between higher DP and circulating inflammatory markers, including interleukin-6 (IL-6), interleukin-8 (IL-8), and the soluble receptor for advanced glycation end products (sRAGE) [18,19]. Current guidance generally supports keeping DP as low as feasible—often below 14–15 cmH_2_O—and monitoring it during surgery as part of a lung-protective ventilation strategy [15].

Among patients receiving extracorporeal membrane oxygenation (ECMO), changes in DP have been reported to correlate with plasma IL-6, sRAGE, TNF-α, and additional mediators [16,19]. Furthermore, ultra-protective ventilation strategies that lower DP during ECMO have been associated with reduced circulating inflammatory biomarkers [20].

In our investigation, lactate values were elevated in the high-DP cohort during the post-CPB period and early ICU phase. Two complementary explanations are plausible: first, inflammatory mediator release related to parenchymal stress; and second, higher intrathoracic pressures that may increase right-ventricular afterload and impair tissue microcirculation. Our study design does not permit a definitive distinction between these mechanisms. Given that postoperative inflammatory markers were comparable between groups, a hemodynamic or microcirculatory explanation may also be plausible. Moreover, the marked inflammatory response inherent to major cardiac surgery may have obscured any subtle DP-related changes in inflammation.

Higher DP has also been linked with an increased need for postoperative respiratory and oxygen support. An initial DP of 13 cmH_2_O or above immediately after induction of general anesthesia has been associated with a 1.92-fold higher likelihood of prolonged postoperative oxygen support [21]. Diminished respiratory system compliance, reflected by a higher DP, has also been associated with longer postoperative mechanical ventilation [22,23]. Among heart-transplant recipients, lower DP has been associated with shorter postoperative ventilation and a briefer ICU stay, although some reports have failed to confirm a direct relationship between lower DP and shorter ICU or hospital stay [15,24]. In our cohort, elevated DP was significantly associated with longer mechanical ventilation, ICU stay, and hospital stay.

Cardiac surgery requiring CPB is accompanied by an intense inflammatory response. In this context, elevated DP may reflect the combined effects of altered lung mechanics, CPB-related pulmonary stress, and perioperative vulnerability rather than a single causal pathway.

Several limitations must be acknowledged. The study was carried out at a single center, was observational and non-randomized, and enrolled a relatively modest number of patients, each of which limits causal inference between perioperative DP and postoperative outcomes and restricts how widely our findings can be extrapolated to the broader cardiac surgical population. Pleural pressures were not recorded with an esophageal balloon catheter; therefore, it is not possible to determine whether the observed elevation in DP arose primarily from parenchymal injury or from reduced chest wall compliance. Several perioperative variables of potential relevance, such as intraoperative fluid balance, transfusion requirements, and detailed postoperative care criteria, were not systematically captured, which raises the possibility of residual confounding. Because DP was sampled only at predefined moments before and after CPB, intraoperative variability may not be fully represented. In addition, because mechanical ventilation was discontinued during CPB according to our institutional protocol, CPB-related atelectasis and derecruitment may have influenced post-CPB respiratory mechanics and postoperative pulmonary outcomes. The chosen DP threshold of 13 cmH_2_O may not be optimal for our population, and PEEP titration was not undertaken, leaving the possible benefit of personalized PEEP in patients with elevated DP unanswered. Although both clinical and experimental evidence supports associations between increasing DP and systemic inflammatory biomarkers such as IL-6, IL-8, and sRAGE, these markers were not measured in our study; therefore, the DP–inflammation interaction could not be analyzed. PPCs were classified using clinical and radiological standards and may therefore be subject to interobserver variability. Because some individual PPC components, such as pneumothorax and pneumonia, occurred in very small numbers, component-level comparisons should be interpreted as exploratory and may be vulnerable to statistical instability and type I error. Lastly, only short-term outcomes, including 30-day mortality, were evaluated, and longer-term respiratory and functional results were not assessed; the link between higher DP and PPCs should accordingly be interpreted as a clinical association rather than as evidence of causation.

## 5. Conclusions

In this prospective observational study of patients undergoing CPB-supported cardiac surgery, an intraoperative DP ≥ 13 cmH_2_O was associated with a higher unadjusted burden of postoperative pulmonary complications and with delayed postoperative recovery, including prolonged mechanical ventilation and longer ICU and hospital stays. However, after adjustment for BMI, CPB time, and age, pre-CPB DP was not independently associated with the composite PPC endpoint. These findings suggest that elevated DP may serve as a clinically useful marker of impaired respiratory mechanics and postoperative vulnerability rather than as an independent causal determinant of PPCs. Larger multicenter prospective studies are needed to validate the optimal DP threshold in cardiac surgery and to determine whether DP-guided ventilatory strategies may improve postoperative pulmonary outcomes.

## Figures and Tables

**Table 1:** Demographic data [*n* (%), mean ± standard deviation, median (min–max)].

	Group I (*n* = 66)	Group II (*n* = 33)	*p*
Gender (F/M)	19/47	13/20	0.287
Age (years)	59.14 ± 10.89	59.58 ± 8.91	0.842
BMI (kg/m^2^)	27.21 ± 4.25	29.45 ± 4.82	0.037 *
ASA III/IV	58/8	25/8	0.122
Smoking	19 (28.79%)	10 (30.30%)	0.876
Bronchodilator use	4 (6.06%)	6 (18.18%)	0.066
FVC	86.09 ± 12.53	82.88 ± 14.43	0.259
FEV1	89.36 ± 15.72	84.12 ± 17.82	0.141
FEV1/FVC	105.08 ± 13.60	105.27 ± 12.45	0.946
EUROSCORE	3 (0–10)	3 (0–8)	0.177
LVEF	51.52 ± 8.45	51.48 ± 9.67	0.805

BMI: body mass index; FVC: forced vital capacity; FEV1: forced expiratory volume in the first second; LVEF: left ventricular ejection fraction. * *p* < 0.05.

**Table 2:** Intraoperative data [mean ± standard deviation, median (min–max)].

	Group I (*n* = 66)	Group II (*n* = 33)	*p*
CPB time (min)	95.05 ± 25.88	101.58 ± 47.19	0.375
Aortic cross-clamp time (min)	67.32 ± 19.87	78.24 ± 36.65	0.057
Anesthesia time (min)	247.23 ± 54.47	258.79 ± 69.03	0.366
Surgical time (min)	217.29 ± 49.19	230.00 ± 64.31	0.278
Pre-CPB DP (cmH_2_O)	10.00 (6.00–12.00)	15.00 (13.00–22.00)	<0.001 *
Post-CPB DP (cmH_2_O)	12.00 (6.00–18.00)	16.00 (13.00–22.00)	<0.001 *

Pre-CPB DP: pre-cardiopulmonary bypass driving pressure; Post-CPB DP: post-cardiopulmonary bypass driving pressure. * *p* < 0.05.

**Table 3:** Perioperative hemodynamic parameters and postoperative infection markers (mean ± standard deviation).

	Group I (*n* = 66)	Group II (*n* = 33)	*p*
Preoperative HR (beats/min)	83.05 ± 16.94	84.82 ± 18.13	0.633
Preoperative MAP (mmHg)	96.57 ± 22.96	98.56 ± 19.00	0.667
Pre-CPB HR	82.71 ± 21.73	84.91 ± 22.10	0.638
Pre-CPB MAP	76.48 ± 15.86	77.55 ± 17.86	0.763
Post-CPB HR	106.41 ± 18.72	102.73 ± 18.85	0.360
Post-CPB MAP	66.06 ± 11.65	64.27 ± 9.44	0.446
Postoperative HR	105.52 ± 16.02	102.85 ± 14.99	0.427
Postoperative MAP	82.75 ± 15.63	80.30 ± 11.90	0.430
WBC	9.03 ± 2.35	8.92 ± 2.02	0.823
Neutrophil count	6.92 ± 2.26	6.73 ± 1.88	0.676
Lymphocyte count	2.11 ± 0.68	2.19 ± 0.65	0.554
NLR	3.62 ± 1.60	3.43 ± 1.97	0.606
CRP	7.17 ± 6.19	6.40 ± 5.27	0.545
ESR	22.73 ± 14.14	25.61 ± 20.61	0.474

HR: heart rate; MAP: mean arterial pressure; WBC: white blood cell count; NLR: neutrophil-to-lymphocyte ratio; CRP: C-reactive protein; ESR: erythrocyte sedimentation rate; Pre-CPB: pre-cardiopulmonary bypass; Post-CPB: post-cardiopulmonary bypass.

**Table 5:** Postoperative pulmonary complications and clinical outcomes [*n* (%), median (min–max)].

	Group I (*n* = 66)	Group II (*n* = 33)	*p*
Pneumothorax	0 (0.00)	3 (9.09)	0.035 *
Pleural effusion	13 (19.70)	14 (42.42)	0.017 *
Pneumonia	4 (6.06)	3 (9.09)	0.429
Atelectasis	12 (18.18)	13 (39.39)	0.022 *
CPAP need	2 (3.03)	9 (27.27)	0.002 *
Prolonged ventilation	5 (7.58)	9 (27.27)	0.011 *
Mechanical ventilation duration (h)	8.25 (4.33–69.00)	16.50 (3.60–123.92)	<0.001 *
ICU stay (h)	48.00 (14.66–190.00)	78.33 (22.60–178.33)	0.004 *
Hospital stay (h)	183.92 (36.33–368.00)	248.00 (58.50–382.66)	0.002 *
30-day mortality	7 (10.61)	5 (15.15)	0.363

CPAP: continuous positive airway pressure; ICU: intensive care unit. Prolonged ventilation was defined as mechanical ventilation lasting more than 24 h. * *p* < 0.05.

**Table 6:** Univariable and multivariable logistic regression analysis of factors associated with postoperative pulmonary complications.

Variable	Univariable OR (95% CI)	*p*	Adjusted OR (95% CI)	*p*
Pre-CPB driving pressure (per 1 cmH_2_O)	1.121 (0.988–1.273)	0.077	1.091 (0.952–1.251)	0.212
Body mass index (per 1 kg/m^2^)	1.069 (0.977–1.170)	0.145	1.052 (0.953–1.162)	0.314
CPB (pump) time (per 1 min)	1.002 (0.990–1.014)	0.726	1.002 (0.990–1.014)	0.792
Age (per 1 year)	1.019 (0.979–1.061)	0.355	1.022 (0.979–1.067)	0.322

Data are odds ratios (OR) with 95% confidence intervals (CI). Multivariable model fitted by binary logistic regression (enter method); *n* = 99, 41 events, 4 predictors (≈10.2 events per variable). Covariates were pre-specified on clinical grounds and limited in number to maintain approximately ten events per variable. Estimates are expressed per 1-unit increase. CPB, cardiopulmonary bypass.

**Table 7:** Association between pre-CPB driving pressure and secondary postoperative outcomes.

Secondary Outcome	Measure (per 1 cmH_2_O)	Estimate (95% CI)	*p*
Prolonged postoperative ventilation	Odds ratio	1.314 (1.101–1.568)	0.003
30-day mortality	Odds ratio	1.141 (0.960–1.357)	0.135
ICU length of stay (days)	β coefficient	0.16 (0.07–0.26)	0.001
Hospital length of stay (days)	β coefficient	0.23 (0.04–0.42)	0.019
Duration of ventilation (hours)	β coefficient	2.07 (0.99–3.15)	<0.001

Binary outcomes (prolonged ventilation and mortality) were analyzed by univariable logistic regression (odds ratio per 1 cmH_2_O). Continuous outcomes were analyzed by linear regression (β coefficient per 1 cmH_2_O); all three were also significant on Spearman correlation (ρ = 0.23, 0.22, and 0.23, respectively; all *p* < 0.05). ICU, intensive care unit.

## Data Availability

The datasets generated and/or analyzed during the current study are available from the corresponding author on reasonable request.
